# Dietary Lipids and Dyslipidemia in Chronic Kidney Disease

**DOI:** 10.3390/nu13093138

**Published:** 2021-09-09

**Authors:** Zdzislaw Kochan, Natalia Szupryczynska, Sylwia Malgorzewicz, Joanna Karbowska

**Affiliations:** 1 Laboratory of Nutritional Biochemistry, Department of Clinical Nutrition, Faculty of Health Sciences, Medical University of Gdansk, 80-210 Gdansk, Poland; zdzislaw.kochan@gumed.edu.pl (Z.K.); natalia.szupryczynska@gumed.edu.pl (N.S.); 2 Department of Clinical Nutrition, Division of Clinical Nutrition and Dietetics, Faculty of Health Sciences, Medical University of Gdansk, 80-210 Gdansk, Poland; sylwia.malgorzewicz@gumed.edu.pl; 3 Department of Nephrology, Transplantology and Internal Medicine, Faculty of Medicine, Medical University of Gdansk, 80-210 Gdansk, Poland; 4 Department of Biochemistry, Faculty of Medicine, Medical University of Gdansk, 80-210 Gdansk, Poland

**Keywords:** chronic kidney disease, dyslipidemia, triglycerides, nutrition, dietary patterns, omega-3 PUFA, fish consumption

## Abstract

The progression of chronic kidney disease (CKD) leads to altered lipid metabolism. CKD patients exhibit high blood triglyceride (TG) levels, reduced concentrations and functionality of high-density lipoproteins (HDL), and elevated levels of atherogenic small, dense, low-density lipoproteins (sdLDL). Disorders of lipid metabolism and other metabolic disturbances place CKD patients at high risk for cardiovascular disease (CVD). Extensive evidence supports the cardioprotective effects of unsaturated fatty acids, including their beneficial effect on serum cholesterol and TG levels. Dietary lipids might therefore be especially important in the nutritional management of CKD. We review current dietary recommendations for fat intake by CKD patients and suggest potential nutritional interventions by emphasizing dietary lipids that might improve the blood lipid profile and reduce cardiovascular risk in CKD.

## 1. Introduction

Chronic kidney disease (CKD) is a gradual loss of kidney function leading to eventual kidney damage and failure that greatly reduces life expectancy [1,2]. Patients with advanced CKD exhibit metabolic abnormalities that include hyperphosphatemia, secondary hyperparathyroidism, hypocalcemia, and dyslipidemia [1,3]. Along with the chronic inflammation, increased oxidative stress, and malnutrition associated with CKD, these abnormalities elevate cardiovascular risk [4,5]. The proportion of CKD patients who die from cardiovascular disease (CVD) increases as their glomerular filtration rate (GFR) declines and kidney disease progresses from GFR category G1 to G5 [6]. CVD is the leading cause of death for people with an estimated GFR (eGFR) < 60 mL/min per 1.73 m^2^ [6]. Patients with end-stage renal disease (ESRD) die of cardiovascular diseases at 10–30 times the rate of the general population [2].

Lipid abnormalities characteristic of CKD include hypertriglyceridemia and low levels of high-density lipoprotein cholesterol (HDL-C); both conditions are major risk factors for CVD [2]. Patients with CKD exhibit a specific fatty acid profile of elevated serum monounsaturated fatty acid (MUFA) concentrations and decreased polyunsaturated fatty acid (PUFA) concentrations [7]. CKD-associated depletion of PUFAs might also increase CVD risk [8]. Dyslipidemia often occurs in the early stages of CKD and worsens as the disease progresses [1]. Dialysis treatment does little to improve these lipid disorders. Though chronic hemodialysis (HD) might reduce triglyceride (TG) levels slightly, peritoneal dialysis (PD) contributes to hypertriglyceridemia because of glucose overload [9]. The risk of cardiovascular complications drops after kidney transplantation compared with dialysis [5], but the fatty acid profile remains altered in renal recipients [7].

Studies indicate that dietary PUFAs might delay the onset of CKD and alleviate CVD as kidney disease progresses [10,11,12,13]. Higher plasma PUFA levels were linked to a lower risk of CKD in a three-year follow-up study of the elderly [10]. Multivariate analysis showed that an increased dietary intake of PUFAs by patients with type 2 diabetes was associated with a lower prevalence of CKD [11]. Supplementation with ω-3 PUFAs for five years, however, did not slow the decline of eGFR in diabetic patients in a longitudinal placebo-controlled study [14]. In another clinical trial, ω-3 PUFAs supplemented for three months did not modify urine albumin excretion in patients with CKD, but significantly lowered serum TG levels, improved arterial stiffness, and reduced systolic blood pressure [13]. In CKD patients on chronic HD, treatment with ω-3 PUFA supplements significantly reduced the number of myocardial infarctions (MI) but had no effect on the number of cardiovascular events [12]. The most recent recommendations by the National Kidney Foundation’s Kidney Disease Outcomes Quality Initiative (KDOQI) advise PUFA supplementation in patients with CKD, but no main authority has yet issued dietary guidelines aimed at increasing PUFA intake [15].

## 2. Dyslipidemia in Chronic Kidney Disease

Lipid disorders associated with CKD are primarily related to the impaired metabolism of circulating TG-rich lipoproteins (TRL): Very-low-density lipoproteins (VLDL), intermediate-density lipoproteins (IDL), and to a lesser extent, chylomicrons. These disorders are also linked to the dysfunction of high-density lipoproteins (HDL) [16,17]. Hypertriglyceridemia develops in CKD patients because the activity of enzymes involved in TRL metabolism is altered in ways that delay TRL’s clearance from plasma. The activity of lipoprotein lipase (LPL, the enzyme that hydrolyzes most TG in TRL) and the liver’s subsequent uptake of TRL remnants are inhibited by apolipoprotein C-III (apoC-III), whose plasma levels are significantly elevated in patients with CKD [17]. Impaired renal apoC-III excretion leads to an increase in circulating apoC-III, which slows the LPL-dependent clearance of plasma TG [17]. Another obstacle to LPL’s effective metabolism of TRL is the unusually high cholesteryl ester content of these lipoproteins at the expense of TG [17]. This might result from the activity of cholesteryl ester transfer protein (CETP), which facilitates the transfer of cholesteryl esters in exchange for TG between lipoproteins and thus increases the amount of cholesteryl esters in VLDL, IDL, and chylomicrons while lowering the TG content in these particles [18]. CETP activity does not increase in CKD [16], but an extended half-life of TRL might promote CETP-mediated remodeling of lipoproteins. Elevated levels of TG in the blood might also result from enhanced hepatic lipogenesis and VLDL secretion in response to high glucose concentrations, especially in patients on PD since standard PD solutions contain high levels of glucose [9].

As kidney disease progresses, CKD-associated dyslipidemia becomes more severe because plasma HDL levels fall [16,17,19,20]. This is mainly a consequence of a gradual decline in the activity of lecithin:cholesterol acyltransferase (LCAT), which is most evident in ESRD [16,19]. Lowering LCAT activity impairs the maturation of the lipid-poor precursors of HDL (pre-β HDL) to spherical HDL particles [19]. Moreover, uremic HDLs are enriched in TG and depleted of cholesteryl esters, as shown in patients with ESRD undergoing dialysis [16]. An increase in TG content with a concomitant decrease in the amount of cholesteryl esters, most probably catalyzed by CETP, destabilizes HDL particles yielding pre-β HDL. Elevated plasma levels of pre-β HDL characterize advanced CKD and ESRD [19,21]. Pre-β HDLs are more rapidly degraded and cleared by the kidney than are mature HDLs, which reduces circulating levels of apolipoprotein A-I (apoA-I), the main structural protein of HDL [19,20,22]. HDL particles in patients on dialysis contain significantly lower amounts of apoA-I than functional HDL since it is replaced by pro-inflammatory serum amyloid A (SAA) [16,23]. Both apoA-I-depleted uremic HDL and pre-β HDL are less efficient than fully functional HDL in promoting cholesterol efflux from macrophages and peripheral tissues [16,24,25,26]. Along with a significant reduction in HDL levels, limited efflux impairs reverse cholesterol transport in patients with advanced stages of CKD [17,18]. ESRD is also associated with other unfavorable changes to the proteome of HDL; for example, HDL particles acquire uremic toxins such as symmetric dimethylarginine (SDMA) but paraoxonase (PON) content and activity decline [16,27]. Together with the altered lipid composition, these changes contribute to HDL losing its antioxidant, anti-inflammatory, and vasoprotective properties [16,22,25,26,27].

Dyslipidemia in CKD also manifests as an increase in plasma levels of small, dense LDL (sdLDL), the particularly dangerous and the most atherogenic fraction of low-density lipoproteins (LDL) [28]. The atherogenic burden in patients with late-stage CKD or ESRD is compounded by higher levels of lipoprotein(a) (Lp(a)) [20,22]. In patients on dialysis, this is probably caused by the decreased catabolism of Lp(a). Elevated levels of Lp(a) are a powerful independent risk factor for atherosclerotic CVD and myocardial infarction in CKD patients [29].

## 3. Digestion, Absorption, and Regulatory Role of Dietary Lipids

Lipids account for more than 30% of the total energy intake in the Western diet [30]. The most abundant dietary lipids are triglycerides, which consist of three fatty acids attached to a glycerol molecule. Ingested TGs are hydrolyzed in the digestive tract, resynthesized in enterocytes, and incorporated into chylomicrons. As part of chylomicrons, TGs are then transported in the blood to body tissues where fatty acids are released by LPL and absorbed. Dietary fatty acids not only serve as a source of energy to the body, but some also regulate metabolism. PUFAs, for example, influence the activity of transcription factors involved in the control of lipid metabolism, such as the sterol regulatory element-binding protein 1 (SREBP-1), the peroxisome proliferator-activated receptor α (PPARα), and the liver X receptor α (LXRα) [31,32,33,34].

SREBP-1c, the predominant isoform of SREBP-1 in metabolic tissues, is mainly involved in the induction of genes associated with fatty acid and TG synthesis [35]. The promoter of SREBP-1c is stimulated by insulin and by LXR [36]. Long-chain PUFAs of the ω-3 series, such as eicosapentaenoic acid (EPA, C20:5 n-3), can modulate the amount of SREBP-1 released into the nucleus by inhibiting proteolytic cleavage of an inactive precursor of SREBP-1 located in the ER membrane [31]. Thus, levels of the active form of this transcription factor are reduced in the presence of EPA [31]. In contrast to saturated fatty acids, unsaturated fatty acids can act as competitive antagonists of LXRα that impair this nuclear receptor binding to DNA and reduce the expression of LXR target genes, including the gene that encodes SREBP-1c [33,34]. In vitro studies have shown that arachidonic acid (AA, C20:4 n-6), EPA, and docosahexaenoic acid (DHA, C22:6 n-3) are potent suppressors of SREBP-1c expression, but the average effect is observed after the administration of linoleic acid (LA, C18:2 n-6) or oleic acid (OA, C18:1 n-9) [33,34]. In vivo experiments have demonstrated that PUFA deficiency induces hepatic expression of SREBP-1c and its target genes, such as the gene encoding glycerol-3-phosphate acyltransferase 1 (GPAT1), the enzyme that initiates the synthesis of TG [37]. This leads to an increase in VLDL secretion and subsequent hypertriglyceridemia [37]. More importantly, studies have shown that dietary supplementation of ω-3 and ω-6 PUFAs (DHA and AA) normalizes plasma triglyceride levels [37].

**Figure 1:**
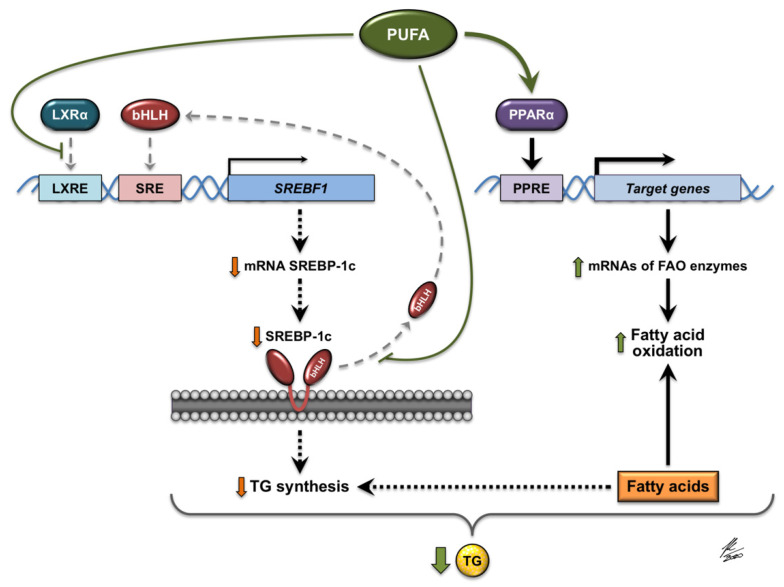
Molecular mechanisms involved in the regulation of gene expression by PUFAs (polyunsaturated fatty acids). PUFAs interfere with the binding of LXRα (liver X receptor α) to DNA and decrease the expression of LXRα target genes, including the *SREBF1* gene that encodes SREBP-1c (sterol regulatory element-binding protein 1c). Moreover, PUFAs inhibit the proteolytic release of the active form of SREBP-1c (bHLH, basic helix-loop-helix) from the membranes of the endoplasmic reticulum, thus preventing the translocation of this transcription factor to the nucleus. The expression of genes activated by SREBP-1c, such as *SREBF1,* and the genes associated with the synthesis of fatty acids and TGs (triglycerides), is therefore diminished. PUFAs also act as natural ligands that activate PPARα (peroxisome proliferator-activated receptor α), which in turn induces the expression of its target genes, such as those encoding the enzymes involved in hepatic oxidation of fatty acids. JK/2020: Author’s signature.

Long-chain PUFAs, especially EPA and DHA, are also potent natural ligands for PPARα and are able to induce a significant increase in the transcription of genes regulated by this nuclear receptor [32]. PPARα is the predominant PPAR subtype in oxidative tissues and, as a transcription factor, it mediates the effects of fatty acids on the expression of several genes associated mainly with fatty acid oxidation [38]. Hepatic PPARα, a master regulator of fatty acid catabolism in the liver, is activated by dietary fatty acids that enter the liver as part of triglycerides and phospholipids in chylomicron remnants, but not by free fatty acids released by lipolysis from adipose tissue [39]. Increased dietary intake of PUFAs might therefore enhance the partitioning of fatty acids to the oxidative pathways in the liver and alter hepatic triglyceride turnover (Figure 1).

## 4. Dietary Approach to Dyslipidemia in CKD Patients

Guidelines for the treatment of CKD patients, including dietary recommendations, are developed by international associations such as Kidney Disease: Improving Global Outcomes (KDIGO) and the National Kidney Foundation’s Kidney Disease Outcomes Quality Initiative (KDOQI) [15,40]. Recommendations for a suitable renal diet vary depending on the CKD stage and further treatment—including HD or PD. The most important elements of the diet are an adequate energy intake of about 25–35 kcal/kg/d, adjusted to maintain normal nutritional status; protein content in the diet, which should decrease to 0.55–0.6 g/kg/d with the loss of kidney function but should increase to 1.0–1.2 g/kg/d once dialysis is initiated; and the restriction of dietary sodium, phosphate, and potassium [15]. The 2020 update of the KDOQI guidelines, which was directed at nutrition in kidney diseases, only mentioned supplementation with long-chain ω-3 PUFAs among its recommendations for fat intake in the management of dyslipidemia in CKD [15], but provided no guidance on the dietary intake of these fatty acids. KDIGO published its previous guidelines for lipid management in CKD in 2013 [40] but recommended only pharmacological lipid-lowering treatment [41]. Subsequent updates have provided no specific dietary recommendations on fat [42]. Comprehensive guidelines for lipid intake in CKD patients therefore remain lacking. The most frequent suggestion is that dietary fat intake in these patients should be the same as recommended for the general population or for patients with other CKD-associated diseases [15,28,40].

The general recommendations for dietary fat intake in patients with lipid disorders have changed insignificantly in recent years and are consistent with the dietary guidelines from the American Heart Association (AHA), the European Society of Cardiology/European Atherosclerosis Society (ESC/EAS), and the National Cholesterol Education Program Therapeutic Lifestyle Changes (NCEP TLC) of the US National Institutes of Health, known also as the Adult Treatment Panel III (ATP III) [43,44,45]. They recommend that total fat intake should provide 25–35% of daily energy, with saturated fatty acids (SFA) restricted to no more than 7%, trans-fatty acids (TFA) to less than 1%, MUFAs up to 20%, and PUFAs up to 10% of the energy from the diet. The amounts of different fatty acids in the diet and their relation to each other are important for generating the best LDL-to-HDL ratio [46]. Clinical and epidemiological studies have examined the importance of the ω-6 to ω-3 fatty acid ratio and the PUFA: MUFA:SFA ratio (PMS ratio) in lipid metabolism, inflammation control, and prevention of cardiovascular diseases [46,47]. These studies recommend an intake of fatty acids in the ratio of ω-6:ω-3 PUFAs of about 5:1 and a PMS ratio range from 1:1:1 to 1:1.3:1 [46,47]. Hypercholesterolemic adults should limit their daily cholesterol intake to 200 mg [43]. These values have also been accepted as the basis for dietary treatment of CKD patients with dyslipidemia [15,40,43,48].

As CKD progresses, the fatty acid composition of the blood lipidome changes: Patients with CKD stages 4–5, on HD or PD, or after kidney transplantation, have elevated serum MUFA levels and lowered levels of long-chain ω-3 and ω-6 PUFAs [7,8,49,50,51]. Factor analyses of serum fatty acids used as indicators of dietary fat quality in two independent Swedish population-based surveys of elderly individuals with CKD stage 3–4 showed that low serum LA and high SFA are strongly associated with metabolic syndrome, insulin resistance, and inflammation [52]. In another study, LA levels in plasma phospholipids of patients on hemodialysis were inversely associated with markers of inflammation and the risk of overall mortality [53]. These findings suggest that a nutritional intervention should be designed to change the dietary intake of fatty acids. In some cases, especially in CKD patients with severe dyslipidemia, ω-3 PUFA supplementation should also be considered [15,54]. CKD patients’ diet might be deficient or improperly balanced because of comorbidities such as gastrointestinal diseases, depression, or dementia; decreased appetite; or socioeconomic circumstances. All can lead to malnutrition and protein-energy wasting (PEW) [2,15,54,55]. Personalized nutrition intervention should improve each patient’s nutritional education and status by adjusting the diet to individual needs.

In the dietary management of dyslipidemia, CKD patients might benefit more from well-balanced dietary patterns than from targeting individual constituents, because of the synergistic effects of different foods and nutrients. Healthy dietary patterns rich in whole grains, vegetables, fruits, legumes, nuts, and fish are associated with a lower incidence of CKD, as shown in a recent meta-analysis of 18 prospective cohort studies [56]. Another study found a consistent association between healthy dietary patterns and lower mortality in patients with CKD [57]. Healthy dietary patterns emphasize nutrient-dense foods with a higher nutritional value than those in standard potassium- and phosphate-restricted renal diets. Patients with CKD should therefore modify the renal diet but take care to avoid hidden sources of potassium and phosphorus, and to use food preparation methods that lower potassium and phosphorus content, such as double boiling or boiling in a large volume of water [58,59,60]. Evidence suggests that certain types of diet are more effective than others in CKD-associated lipid disorders: For example, the Mediterranean diet, Dietary Approaches to Stop Hypertension (DASH), and vegetarian diets have been shown to improve these patients’ lipid profiles [15,61,62,63,64]. All of these diets are rich in vegetables, legumes, fruits, grains, and unsaturated fats. The Mediterranean diet is based on fresh and seasonal vegetables, fruits, whole-wheat products, olive oil, poultry, and fish [65]. DASH emphasizes vegetables, fruits, no- or low-fat dairy, whole grains, nuts, and legumes, but restricts the consumption of sodium, cholesterol, saturated fat, red and processed meat, sweets, and added sugars [66]. Raw vegetables, fruits, and whole grains provide soluble and insoluble fiber, and nutrients such as vitamins, antioxidants, and trace elements. Vegetables and fruits also provide an alkali load—foods high in potassium, magnesium, and calcium increase the production of alkali precursors [58]. A plant-based dietary approach to stage 3 CKD has shown large reductions in blood TG levels in a case study [64]. Eating more fruits and vegetables improved metabolic acidosis, slowed eGFR declines, and reduced blood pressure in patients with CKD stage 3 in a three-year intervention trial [67]. Another recent study of CKD patients found that magnesium modified the interaction between blood lipids and atherosclerotic CVD: Hypertriglyceridemia increased carotid intima–media thickness (cIMT) when magnesium levels were low [68]. The increased dietary intake of magnesium might thus contribute to the atheroprotective effects of plant-based diets in patients with CKD-associated dyslipidemia. Patients with advanced CKD and on dialysis should, however, restrict dietary potassium to less than 3 g/d [58]. These patients should be advised how to adjust the Mediterranean diet, DASH, or a vegetarian diet to reduce the potassium load. Dietary restriction of phosphorus is also highly recommended in CKD to prevent retention of the minerals and hyperphosphatemia associated with bone mineral disorders and vascular calcification [15]. Food’s phosphorus burden can be roughly estimated by using the phosphorus-to-protein ratio (PPR), which would help patients select foods that are rich in protein but low in phosphorus [59]. This is especially important for HD and PD patients, who require a high-protein diet (1.0–1.2 g of protein/kg/d) while limiting phosphorus [15]. The gastrointestinal absorption rate of phosphate from protein-rich foods such as meat, fish, dairy, and eggs, which contain organic phosphorus, is 40–60% [59,69]. In plant foods such as legumes, nuts, and whole grains, most organic phosphorus is bound to phytate. Humans lack phytase, so plant phosphorus is less absorbable, with a bioavailability of 20–50% [69]. The inorganic phosphorus added to processed foods as preservatives, stabilizers, and taste enhancers has the highest bioavailability of 90–100% [59,69]. A crossover trial starkly demonstrated how bioavailability affects the absorption of phosphorus from food. Patients with CKD stage 3–4 received a diet based on meat and dairy (meat/dairy diet) or a diet based on grains and soy (vegetarian diet). After one week, the patients on the meat/dairy diet had significantly higher levels of serum phosphorus and fibroblast growth factor-23 (FGF23), but lower levels of parathyroid hormone (PTH), than patients on the vegetarian diet, though both diets contained similar amounts of phosphorus [70].

Unsaturated fat rich in ω-3 PUFA might also improve dyslipidemia in CKD [13,15]. Oily fish, vegetable oils, nuts, flaxseed, and chia seeds are particularly valuable sources of ω-3 PUFAs and should be considered as part of a healthy diet for CKD patients [71]. Two exploratory factor analyses that assessed food consumption and derived a posteriori food patterns in a CKD population placed fish in the pattern described as “mixed” [72] or “unhealthy” [73], along with red meat and poultry, probably because of these foods’ high protein content. A more recent systematic review and meta-analysis of 17 observational studies involving 149,958 participants that assessed the association between common dietary patterns and CKD, however, found that a healthy dietary pattern associated with a lower risk of CKD was characterized by a high intake of vegetables, fruits, fish, low-fat milk, and whole grains [74].

## 5. Food Sources of Fatty Acids for Patients with CKD

Fat is one of the main macronutrients and provides more energy per gram than any other nutrient. Dietary fats can be sourced from animals and plants. Animal and plant lipids are mixtures in different proportions of glycerol esters of many different fatty acids—saturated and unsaturated [71]. Fats from plants, especially vegetable oils, contain essential unsaturated fatty acids, mainly LA [71]. Plant foods rich in ω-6 PUFAs, such as corn oil or sunflower oil and seeds, are therefore usually recommended for patients with kidney diseases. Another plant-derived essential fatty acid, α-linolenic acid (ALA, C18:3 n-3), belongs to the ω-3 series of PUFAs. Several studies have found evidence for the cardioprotective benefits of ω-3 PUFAs [75,76]. The best plant sources of ALA include flaxseed, perilla seeds, chia seeds, walnuts, and soybeans (Table 1) [71,77]. Typical Western diets contain far greater amounts of ω-6 PUFAs than of ω-3 PUFAs [78]. Patients are therefore advised to increase their intake of ω-3 PUFAs and to use oils rich in ALA, such as perilla or flaxseed oil, rather than corn or sunflower oil [79,80]. Flaxseed oil has the additional advantage that it does not contain potassium, in contrast to flaxseeds [71]. A better range of fatty acids can often be obtained when different vegetable oils are mixed, for example, rice bran or sunflower oil with mustard or groundnut oil [81]. Canola oil and olive oil are also well balanced [82], but coconut oil, coconut milk, and other products containing coconut fat should be restricted because of their high SFA content [81]. Plant products such as partially hydrogenated vegetable oils and hard margarines, as well as foods such as sweet bakery products, French fries, and fast foods, contain many different TFAs, which adversely affect plasma lipids and lipoproteins [83]. TFAs might also increase the risk of coronary heart disease, so foods that contain TFAs should be excluded from the diet [44,81,82].

Animal products such as lard, bacon, fatty red meat, and high-fat dairy products—including butter, cream, whole milk, and most types of cheeses—are rich in SFA and cholesterol: A high intake increases cardiovascular risk, so their consumption should be limited [84]. Animal foods vary in their fat content and composition, and therefore affect kidney function differently. In contrast to high-fat dairy products, consumption of low-fat dairy foods such as no- or low-fat milk and low-fat yogurt is associated with a lower risk of CKD [85]. One reason for this could be the low SFA content in these products [71]. A recent systematic review of 21 prospective cohort studies with at least three years of follow-up found no significant associations with kidney function and poultry consumption, but the consumption of red or processed meat was associated with a higher risk of CKD [63]. Poultry has a higher proportion of unsaturated fatty acids to SFAs than red meat, and thus has a more favorable fatty acid composition [71]. Eggs are another animal food that contains considerable amounts of unsaturated fat, but researchers remain concerned about their potential effect on blood lipids and CVD risk because of the yolk’s high cholesterol content (1080 mg/100 g; 184 mg of cholesterol per 1 large (17 g) egg yolk [71]) [86]. The 2019 ESC/EAS guidelines for the management of dyslipidemia advocate limiting dietary cholesterol to less than 300 mg daily, particularly for individuals with elevated plasma cholesterol levels [44]. ATP III recommends no more than 200 mg/d of dietary cholesterol for adults with hypercholesterolemia [43]. Patients with CKD, however, are hypertriglyceridemic, not hypercholesterolemic—especially in more advanced stages, since plasma levels of total cholesterol, LDL-cholesterol (LDL-C), and HDL-C decrease as kidney disease progresses [19,68]. Few studies have examined the effects of eggs on the lipid profile in uremic patients and their results are inconclusive [87,88]. The current KDOQI guidelines provide no recommendations on egg consumption [15], but eggs are one of the most nutrient-dense foods, are naturally low in sodium, and are a rich source of high-quality protein [71], so they should not be excluded from CKD patients’ diet.

Oily fish is the main dietary source of health-promoting long-chain ω-3 PUFAs, with marine species such as herring, mackerel, salmon, and sardines being particularly high in EPA and DHA (Table 2). Large amounts of long-chain ω-3 PUFA (usually more than 20 g/100 g) are also found in oils extracted from fish bodies or livers. Fish liver oil, however, is rich in retinol: 100 g of cod liver oil contains 30,000 µg of preformed vitamin A [71]. Patients with CKD should carefully monitor their intake of preformed vitamin A, because of the risk of pathological accumulation of retinol resulting from elevated levels of serum retinol-binding protein 4 (RBP4) in renal failure [89,90]. EPA and DHA are also present at relatively high concentrations of no less than 0.5 g/100 g in rainbow trout, tuna, swordfish, pompano, and certain crustaceans and shellfish [71]. Most fish and other seafoods are a rich source of long-chain ω-3 PUFA, but are also naturally low in cholesterol, with less cholesterol content than poultry and red meat [71].

## 6. Omega-3 PUFA in CKD

The advantages of dietary ω-3 PUFA in targeting CKD were demonstrated when significant negative associations were found between the consumption of fish, ALA, or other long-chain PUFAs of the ω-3 series and the incidence of CKD [91,92]. Serum phospholipid long-chain ω-3 PUFA levels are also inversely related to the risk of sudden cardiac death in the first year of HD [8]. The Institute of Medicine recommends the same dietary intake of ω-3 PUFA for HD patients without dyslipidemia as the recommended intake of ALA for the general population: An adequate intake (AI) for ALA is 1.6 g/d for men and 1.1 g/d for women [47]. Up to 10% of the AI for ALA can be consumed as EPA or DHA [47].

Supplementation of ω-3 PUFA in patients undergoing HD might reduce cardiovascular mortality, though it appears that a dose of at least 3 g/d is required to achieve the beneficial effect [93]. A two-year randomized, double-blind, and placebo-controlled intervention trial found that daily supplementation with 1.7 g of ω-3 PUFA did not affect all-cause mortality or the number of cardiovascular events in CKD-HD patients with established CVD, but did significantly reduce the incidence of MI in these patients [12]. A recent systematic review and meta-analysis of 60 randomized controlled trials (RCTs) evaluating the effect of ω-3 PUFA supplementation on cardiovascular and all-cause mortality in patients with CKD found low-certainty evidence that supplementation with ~3 g of ω-3 PUFA daily might reduce the risk of cardiovascular mortality in HD and kidney transplant patients, but found no significant differences in all-cause mortality for patients in CKD stages 1–5, on dialysis, or after transplantation [93]. None of the studies in this meta-analysis examined the effects of dietary modification such as increasing the intake of foods rich in ω-3 PUFA, yet the cardiovascular benefit of supplementation with ω-3 PUFA was greater in patients on HD than in the general population [93]. This is especially important because lipid-lowering therapies barely reduce cardiovascular risk in patients with ESRD once dialysis is initiated [94,95]. Statin therapy was associated with higher rates of major adverse cardiac and cerebrovascular events (MACCEs), and with acute myocardial infarction in young adult ESRD patients receiving dialysis [95]. In another study, rosuvastatin failed to reduce rates of myocardial infarction, stroke, or death from cardiovascular causes in middle-aged and older ESRD patients undergoing maintenance HD [94]. Statins are less effective in reducing CVD risk for patients on dialysis probably because of chronic inflammation and imbalanced calcium and phosphate levels. These are common comorbidities in advanced CKD that lead to adverse outcomes. Long-chain PUFAs in the ω-3 series exhibit anti-inflammatory properties. Raising blood ω-3 PUFA levels by fish oil supplementation significantly reduced C-reactive protein (CRP) levels in HD patients in a pilot study [96]. A more recent prospective randomized double-blind trial in CKD patients undergoing HD found that supplementation with 2.4 g/d of ω-3 PUFA for 12 weeks significantly lowered the levels of inflammatory markers such as CRP, interleukin-6 (IL-6), and tumor necrosis factor α (TNFα) [97]. The anti-inflammatory effect of ω-3 PUFA was confirmed for ESRD in a systematic review and meta-analysis of 13 RCTs that showed fish oil significantly decreased serum CRP and reduced cardiovascular events in patients on maintenance HD [98].

Supplementation with long-chain ω-3 PUFA should be considered for CKD patients suffering from lipid disorders. Long-chain ω-3 PUFA at the dose of ~2 g/d is suggested to lower blood TG levels in patients with CKD stages 3–5, but for patients on PD or HD, a dose of 1.3–4 g/d is recommended to reduce TG and LDL-C and raise HDL-C levels in the blood [15,99]. Recent ω-3 PUFA supplementation trials, however, have yielded discordant results. In a single-blind randomized crossover study, co-supplementation of ω-3 PUFA and α-tocopherol (920 mg EPA, 760 mg DHA, and 8 mg α-tocopherol daily) for four weeks did not affect lipid profiles in 22 patients on chronic HD [100]. Similarly, a 12-week randomized double-blind placebo-controlled clinical trial of ω-3 PUFA supplementation (1.25 g/d of ω-3 PUFAs containing 600 mg EPA, 300 mg DHA, and 350 mg of other ω-3 PUFAs) and combined supplementation of ω-3 PUFA and vitamin E in two groups of 30 chronic HD patients showed no influence on the serum lipid profile [101]. A study on supplementation with fish oil capsules (1.28 g/d of ω-3 PUFAs, EPA + DHA) for 12 weeks increased HDL-C but failed to improve hypertriglyceridemia in HD patients [102]. Yet, a systematic review and meta-analysis of 13 RCTs showed that fish oil supplementation improved hypertriglyceridemia in patients on maintenance HD: Fish oil significantly reduced serum TG levels without substantial changes in serum total cholesterol and LDL-C levels [98]. In another systematic review and meta-analysis of 13 RCTs, a reduction in serum TG and total cholesterol, with a concomitant increase in HDL-C levels, was achieved in HD and PD patients by four weeks to 12 months of daily supplementation with fish oil that contained from 0.9 g to 3 g of ω-3 PUFA [99]. Fish oil, however, showed no significant effect on LDL-C levels [99]. The suggested intake of fish oil to lower serum TG levels for patients on dialysis was estimated at more than 1 g per day [99]. In another randomized double-blind controlled trial in HD patients, daily consumption of 6 g of flaxseed oil rich in ALA for eight weeks significantly reduced serum TG levels [79]. A recent randomized, double-blind, and placebo-controlled clinical trial in patients with pre-dialysis CKD found that supplementation with 3.7 g/d of ω-3 PUFAs (EPA + DHA) for 12 weeks lowered serum TG levels from 163.5 mg/dl to 117.5 mg/dl [13]. Similarly, a significant decrease in serum TG from 1.97 ± 0.98 mmol/l at baseline to 1.65 ± 0.76 mmol/l was observed in non-fasting CVD patients treated with chronic HD for at least six months after three months of supplementation with ω-3 PUFA (1.7 g of ω-3 PUFAs daily, 45% of EPA, and 37.5% of DHA), although total cholesterol, LDL-C, and HDL-C were not affected [103].

The effects of consuming fish in these HD patients were more promising: High consumption of fish—two to three meals a week or daily meals—significantly raised levels of EPA and DHA in serum phospholipids, and increased plasma levels of HDL-C [103]. Moreover, serum TG decreased from 1.99 ± 1.1 mmol/l to 1.56 ± 0.61 mmol/l by increasing fish intake [103]. Increased consumption of ω-3 PUFA might therefore improve the lipid profile in patients on dialysis. In CKD patients treated with HD or PD, 14 weeks of a dialysis diet modified according to dietary guidelines for dyslipidemia, mainly by maintaining a PMS ratio as close as possible to 1:1:1, normalized total cholesterol and LDL-C levels [104]. The beneficial effects of the dietary intervention were more pronounced in patients on HD; the main problem in achieving an improved lipid profile was adherence to the diet [104].

## 7. Inclusion of Fish in the Diet of Patients with Advanced CKD

Studies in the general population have shown that low or moderate fish intake—30–60 g/d or one to two servings a week—protects against CVD risk [105,106]. Moderate consumption of oily fish was associated with a reduced risk of heart failure (HF) in middle-aged and older women: Those consuming two servings of oily fish a week had a 30% lower rate of HF events than those who did not [107]. A large prospective study conducted in Japan, where more than 90% of adults eat fish at least once a week, demonstrated that a higher fish intake of 8 times a week or 180 g/d was associated with a substantially reduced risk of coronary heart disease (CHD), primarily non-fatal coronary events, among middle-aged women and men [108]. The most recent meta-analysis evaluating the effect of fish consumption on the incidence of CHD and mortality in the general adult population found that eating 20 g/d more fish was associated with a 4% reduction of CHD prevalence and death rate [106]. An intake of 60 g of fish a day was proposed as optimal to decrease the death rate from CHD [106].

Many studies have linked fish consumption to a reduction in cardiovascular risk in the general population, but the amount of fish required to improve dyslipidemia and to reduce cardiovascular events and mortality in CKD patients has not yet been established. In the Atherosclerosis Risk in Communities (ARIC) study, with a median follow-up of 23 years, higher fish and seafood consumption was significantly associated with a lower risk of incident CKD when tested for this trend [85]. A study of habitual fish consumption in a cohort of American HD patients, however, found that nearly 70% of patients on HD consumed far less than the AHA recommends for individuals at high cardiovascular risk: Eating ≤1 serving/week instead of at least one or two servings a week [109,110]. The DIET-HD (DIETary intake, death and hospitalization in adults with end-stage kidney disease treated with HemoDialysis) study, a multinational prospective cohort study of 8110 HD patients, also found that 31% of participants did not consume any fish on a weekly basis, 46% consumed ≤1 serving a week, and 23% ≥ 2 servings each week [111]. The median intake of ω-3 PUFA was therefore 1.2 (0.3–2.4) g/week [111], which is far lower than the minimum recommended value to prevent cardiovascular risk. Consistent evidence indicates that consumption of ~250 mg/d or 1.75 g/week of marine ω-3 PUFAs, EPA + DHA, equivalent to ~1–2 servings/week of oily fish, is required to reduce the risk of cardiac mortality [112]. Consequently, no association between dietary intake of ω-3 PUFAs and cardiovascular and all-cause mortality was detected in HD patients in a median follow-up of 2.7 years [111]. Patients on HD are at high CVD risk, and it has been estimated that they require a higher intake of marine ω-3 PUFA of at least 1 g/d or 7 g/week to reduce this risk [99]. Thus, it is reasonable to assume that an adequate amount of ω-3 PUFAs for patients with advanced CKD can be obtained by eating 2–3 servings/week of oily fish such as herring, mackerel, salmon, or sardines, or 3–6 servings/week of bass, drum, pompano, rainbow trout, swordfish, or tuna (Table 2). One serving is equivalent to a 150 g fillet of fish. The benefits of dietary fish intake in advanced CKD have been shown in a prospective study of reported fish consumption and mortality risk that followed a cohort of HD and PD patients for three years. The study demonstrated that those who ate fish had a mortality rate about 50% lower than patients who did not [113]. Furthermore, higher red blood cell DHA levels, which are determined primarily by dietary consumption of oily fish, have been associated with lower all-cause mortality in Japanese HD patients who were followed for 10 years [114]. Fish is an important source of ω-3 PUFAs and protein, but it is also rich in phosphorus, and farmed fish fed on animal-derived proteins contain higher amounts of phosphorus than wild fish [71]. Therefore, fish with a lower PPR should be selected for consumption, such as wild Atlantic salmon or pompano [71]. Bones should also be removed to reduce phosphorus intake. Some fish contain high amounts of mercury (Hg), so large, long-lived species such as swordfish, shark, marlin, and tilefish should be avoided or eaten less often [112,115]. Table 2 shows the composition of common fish to help make the right choices.

Health benefits can also be affected by how the fish are cooked. Most concerns are related to frying, which can change the fatty acid composition of fish by the addition of oils or other fats [116]. Fried fish, especially in batter, contains high amounts of fat and has a high ω-6:ω-3 PUFA ratio [116]. The associations between usual dietary fish intake and cardiac structure, function, and hemodynamics were investigated among 5073 older women and men in the Cardiovascular Health Study (CHS) [117]. It found the intake of tuna or other broiled or baked fish to be associated with improved cardiac hemodynamics, but fried fish was linked to left ventricular structural abnormalities indicating systolic dysfunction and probable coronary atherosclerosis [117]. Similarly, the consumption of more than one serving a week of broiled or baked fish was associated with a lower risk of incident HF in a cohort of 84,493 postmenopausal women followed for 10 years, but fried fish consumption was associated with an increased HF risk [118]. A recently published multicenter prospective cohort study with a 25-year follow-up reported a marginally significant inverse association between non-fried fish consumption and CKD incidence in young American adults, implying that non-fried fish consumption might be beneficial in the primary prevention of CKD [92]. Further studies are needed to elucidate how fish-preparation methods influence the health effects of dietary fish consumption, but current evidence strongly suggests that baking or broiling are the healthiest methods of cooking fish, and that frying should be avoided.

## 8. Summary and Conclusions

Kidney dysfunction is associated with changes in the levels and composition of circulating lipids and lipoproteins that lead to a more atherogenic lipid profile. Long-chain ω-3 PUFAs are potent regulators of lipid metabolism, so dietary fatty acids might thus influence the risk and progression of cardiovascular disease in patients with CKD. Clinical trials have demonstrated that a daily intake of at least 1 g of EPA + DHA might improve dyslipidemia in patients on dialysis, emphasizing the importance of eating foods rich in long-chain ω-3 PUFAs. Prospective studies have shown the protective effect of fish consumption on the survival of dialysis patients. Apart from long-chain ω-3 PUFAs, elements such as vitamin D, B vitamins, and selenium might enhance the beneficial effects of consuming fish. Oily fish and fish oils warrant special consideration in the dietary management of CKD-associated dyslipidemia. Observational studies have shown that most patients on dialysis do not consume any fish on a weekly basis or consume less than is required to obtain enough ω-3 PUFAs to prevent cardiovascular disease. Patients with CKD should therefore be encouraged to increase fish consumption. Nutritional education of CKD patients by professionals is advised to individualize the diet and to improve the patients’ food choices to favor unsaturated fats, especially long-chain ω-3 PUFAs from carefully chosen species of oily fish, instead of saturated fats from red and processed meat. Patients with advanced CKD are at substantial risk for malnutrition, so they most likely benefit from well-balanced healthy dietary patterns such as the Mediterranean diet, DASH, or plant-based diets that have been shown to improve the lipid profile and to reduce mortality in people with kidney disease, which might thus complement pharmacological lipid-lowering therapies.

## Figures and Tables

**Table 1:** Nutritional composition of nuts, seeds, and vegetable oils.

Product ^1^	Energy	Total Fat	SFA	MUFA	PUFA	ALA	K	P	Protein	PPR	Servings
Nuts and Seeds											
Flaxseeds	534	42.2	3.7	7.5	28.7	22.80	813	642	18.3	35.1	0.4
Chia seeds	486	30.7	3.3	2.3	23.7	17.80	407	860	16.5	52.0	0.4
Walnuts	654	65.2	6.1	8.9	47.2	9.08	441	346	15.2	21.3	0.9
Hemp seeds	553	48.8	4.6	5.4	38.1	8.68	1200	1650	31.6	52.2	0.9
Pecan nuts	691	72.0	6.2	40.8	21.6	0.99	410	277	9.2	30.2	8.1
Pistachio nuts	560	45.3	5.9	23.3	14.4	0.29	1020	490	20.2	24.3	27.7
Macadamia nuts	718	75.8	12.1	58.9	1.5	0.21	368	188	7.9	23.8	38.8
Pumpkin seeds	559	49.1	8.7	16.2	21.0	0.12	809	1230	30.2	39.3	66.7
Pine nuts	673	68.4	4.9	18.8	34.1	0.11	597	575	13.7	42.0	71.4
Hazelnuts	628	60.8	4.5	45.7	7.9	0.09	680	290	15.0	19.4	92.0
Cashew nuts	553	43.9	7.8	23.8	7.8	0.06	660	593	18.2	32.5	–
Peanuts	563	48.8	6.4	25.6	14.9	0.02	690	380	25.2	15.1	–
Brazil nuts	659	67.1	16.1	23.9	24.4	0.02	659	725	14.3	50.6	–
Almonds	579	49.9	3.8	31.6	12.3	0.00	733	481	21.2	22.7	–
Oils											
Flaxseed oil	884	100	9.0	18.4	67.8	53.37	0	1	0.11	–	0.3
Walnut oil	884	100	9.1	22.8	63.3	10.40	0	0	0	0	1.4
Canola oil	884	100	7.4	63.3	28.1	9.14	0	0	0	0	1.6
Soybean oil	884	100	15.7	22.8	57.7	6.79	0	0	0	0	2.2
Mustard oil	884	100	11.6	59.2	21.2	5.90	0	0	0	0	2.5
Rice bran oil	884	100	19.7	39.3	35.0	1.60	0	0	0	0	9.3
Corn oil	900	100	12.9	27.6	54.7	1.16	0	0	0	0	12.8
Avocado oil	884	100	11.6	70.6	13.5	0.96	0	0	0	0	15.5
Olive oil	884	100	13.8	73.0	10.5	0.76	1	0	0	0	19.5
Sesame oil	884	100	14.2	39.7	41.7	0.30	0	0	0	0	49.4
Cottonseed oil	884	100	25.9	17.8	51.9	0.20	0	0	0	0	74.1
Safflower oil	884	100	7.5	75.2	12.8	0.10	0	0	0	0	–
Coconut oil	892	99.1	82.5	6.3	1.7	0.02	0	0	0	0	–
Sunflower oil	884	100	10.3	19.5	65.7	0.00	0	0	0	0	–
Peanut oil	884	100	16.9	46.2	32.0	0.00	0	0	0	0	–

^1^ Based on data from FoodData Central [71]. Energy [kcal/100 g]; Total fat [g/100 g]; SFA, saturated fatty acids [g/100 g]; MUFA, monounsaturated fatty acids [g/100 g]; PUFA, polyunsaturated fatty acids [g/100 g]; ALA, α-linolenic acid [mg/100 g]; K, potassium [mg/100 g]; P, phosphorus [mg/100 g]; Protein [g/100 g]; PPR, phosphorus-to-protein ratio [mg/g]; Servings, the number of 25 g servings of nuts and seeds or the number of tablespoons (13.5 g) of oil to provide 2 g of ω-3 PUFA (ALA).

**Table 2:** Nutritional composition of fish, mollusks, crustaceans, and fish oils.

Product ^1^	Energy	Total Fat	SFA	MUFA	PUFA	EPA	DHA	CH	K	P	Protein	PPR	Servings
Fish													
Mackerel, Atlantic	205	13.9	3.26	5.46	3.35	0.90	1.40	70	314	217	18.6	11.7	2.0
Anchovies	210	9.7	2.20	3.77	2.56	0.76	1.29	85	544	252	28.9	8.7	2.3
Salmon, Atlantic, farmed	208	13.4	3.05	3.77	3.89	0.86	1.10	55	363	240	20.4	11.8	2.4
Herring, Atlantic	158	9.0	2.04	3.74	2.13	0.71	0.86	60	327	236	18.0	13.1	3.0
Salmon, Atlantic, wild	142	6.3	0.98	2.10	2.54	0.32	1.12	55	490	200	19.8	10.1	3.2
Sardines	185	10.4	2.68	4.82	2.11	0.53	0.86	61	341	366	20.9	17.5	3.3
Tuna, bluefin	144	4.9	1.26	1.60	1.43	0.28	0.89	38	252	254	23.3	10.9	4.0
Tuna, white	128	3.0	0.79	0.78	1.11	0.23	0.63	42	237	217	23.6	9.2	5.4
Swordfish	144	6.7	1.61	3.00	1.15	0.11	0.65	66	418	255	19.7	13.0	6.2
Trout, rainbow, farmed	141	6.2	1.38	1.98	1.51	0.22	0.52	59	377	226	19.9	11.3	6.4
Bass, freshwater	114	3.7	0.78	1.43	1.06	0.24	0.36	68	356	200	18.9	10.6	7.8
Trout, rainbow, wild	119	3.5	0.72	1.13	1.24	0.17	0.42	59	481	271	20.5	13.2	8.0
Pompano	164	9.5	3.51	2.59	1.14	0.18	0.39	50	381	195	18.5	10.6	8.2
Drum, freshwater	119	4.9	1.12	2.19	1.15	0.23	0.29	64	275	180	17.5	10.3	9.0
Pollock, Atlantic	92	1.0	0.14	0.11	0.48	0.07	0.35	71	356	221	19.4	11.4	11.1
Catfish, channel, wild	95	2.8	0.72	0.84	0.87	0.13	0.23	58	358	209	16.4	12.8	12.8
Carp	127	5.6	1.08	2.33	1.43	0.24	0.11	66	333	415	17.8	23.3	13.3
Flatfish (flounder and sole)	70	1.9	0.44	0.54	0.37	0.14	0.11	45	160	252	12.4	20.3	19.0
Pollock, Alaska	76	0.8	0.15	0.10	0.26	0.08	0.16	61	331	190	17.2	11.1	19.9
Halibut	91	1.3	0.29	0.47	0.29	0.07	0.13	49	435	236	18.6	12.7	24.1
Cod, Atlantic	82	0.7	0.13	0.09	0.23	0.06	0.12	43	413	203	17.8	11.4	25.4
Pike, northern	88	0.7	0.12	0.16	0.20	0.03	0.07	39	259	220	19.3	11.4	43.6
Tilapia	96	1.7	0.59	0.50	0.36	0.01	0.09	50	302	170	20.1	8.5	51.3
Catfish, channel, farmed	119	5.9	1.31	2.57	1.12	0.02	0.06	55	302	204	15.2	13.4	63.1
Mollusks and crustaceans													
Squid	92	1.4	0.36	0.11	0.52	0.15	0.34	233	246	221	15.6	14.2	9.6
Crab, blue	87	1.1	0.22	0.19	0.39	0.17	0.15	78	329	229	18.1	12.7	14.6
Mollusks, clam	86	1.0	0.19	0.12	0.19	0.04	0.06	30	46	198	14.7	13.5	43.6
Shrimps	85	0.5	0.10	0.09	0.15	0.03	0.03	161	264	214	20.1	10.6	76.5
Fish oils													
Cod liver oil ^#^	902	100	22.6	46.7	22.0	6.90	11.00	570	0	0	0	0	0.8 *
Fish oil	902	100	21.3	56.6	15.6	6.27	4.21	766	0	0	0	0	1.4 *

^1^ Based on data from FoodData Central [71]. Energy [kcal/100 g]; Total fat [g/100 g]; SFA, saturated fatty acids [g/100 g]; MUFA, monounsaturated fatty acids [g/100 g]; PUFA, polyunsaturated fatty acids [g/100 g]; EPA, eicosapentaenoic acid [mg/100 g]; DHA, docosahexaenoic acid [mg/100 g]; CH, cholesterol [mg/100 g]; K, potassium [mg/100 g]; P, phosphorus [mg/100 g]; Protein [g/100 g]; PPR, phosphorus-to-protein ratio [mg/g]; Servings, the number of 150 g servings of fish, mollusks, or crustaceans per week to provide an average of 1 g/day of the marine ω-3 PUFAs (EPA + DHA); * the number of tablespoons (13.5 g) of oil to provide 2 g of marine ω-3 PUFAs (EPA + DHA); ^#^ Cod liver oil is not recommended for CKD patients because of its high content of retinol (preformed vitamin A).

## Data Availability

Not applicable.
